# Dynamic through-silicon-via filling process using copper electrochemical deposition at different current densities

**DOI:** 10.1038/srep46639

**Published:** 2017-04-19

**Authors:** Fuliang Wang, Zhipeng Zhao, Nantian Nie, Feng Wang, Wenhui Zhu

**Affiliations:** 1 State Key Laboratory of High Performance Complex Manufacturing, Changsha 410083, China; 2 School of Mechanical and Electrical Engineering, Central South University, Changsha 410083, China

## Abstract

This work demonstrates the dynamic through-silicon-via (TSV) filling process through staged electrodeposition experiments at different current densities. Different morphologies corresponding to TSV filling results can be obtained by controlling the applied current density. Specifically, a low current density (4 mA/cm^2^) induces seam defect filling, a medium current density (7 mA/cm^2^) induces defect-free filling, and a high current density (10 mA/cm^2^) induces void defect filling. Analysis of the filling coefficient indicates that the effect of current density on the TSV filling models is triggered by the coupling effect of consumption and diffusion of additives and copper ions. Further, the morphological evolution of plating reveals that the local deposition rate is affected by the geometrical characteristics of the plating.

Through-silicon-via (TSV) is a promising three-dimensional (3D) packaging technology due to its advantages of high performance, reduction in packaging volume, low power consumption, and multi-functionality^1^,^2^,^3^,^4^. During the TSV process, the via filling step—which is commonly performed using copper electrochemical deposition (ECD)—accounts for almost 40% of the total cost^5^. As the core and critical technologies of TSV, defect-free filling with minimization of process time and cost has attracted much attention^6^.

Currently, the research on TSV filling mainly focuses on condition optimization and the filling mechanism. To achieve defect-free TSV filling, the specific additives in the plating solution, *i.e.*, chloride ion, suppressor, and accelerator were intensively investigated^7^,^8^,^9^,^10^,^11^. The optimum electrodeposition parameters corresponding to specific additives and via structure have been widely studied^12^,^13^,^14^,^15^,^16^,^17^. Among these parameters, the effect of current density on the filling morphology is particularly pronounced. Although specific additives effectively allow for defect-free filling, the required steps are complex, deposition rate is low, and associated costs are high. To overcome the defects of the additive-assisted filling method, the electroplating current waveforms such as pulse current, pulse reverse current, periodic pulse reverse current, and multiple steps current were extensively studied^18^,^19^,^20^,^21^,^22^,^23^. At the same time, several mechanisms have been invoked to explain the defect-free filling process, such as the traditional levelling model, convection-dependent adsorption model, time-dependent transport-adsorption model, and curvature enhanced accelerator coverage model^22^.

Dynamic TSV filling is important for all aspects of research on TSV filling. It is the basis for the optimization of the conditions^12^ and reflects the mechanism of the filling models^24^. However, existing studies have only focused on the filling results obtained at specific moments. A continuous and comprehensive dynamic TSV filling process was seldom systematically investigated.

In this study, we demonstrate the TSV dynamic filling process through staged electrodeposition experiments at different current densities. The optimum current density to achieve defect-free filling was obtained. The effect of current density on the filling models is discussed. Further, the morphological evolution of the plating revealed that the local deposition rate was affected by the geometrical characteristics of the plating.

## Results

### TSV filling results at different current densities

**Figure 1:**
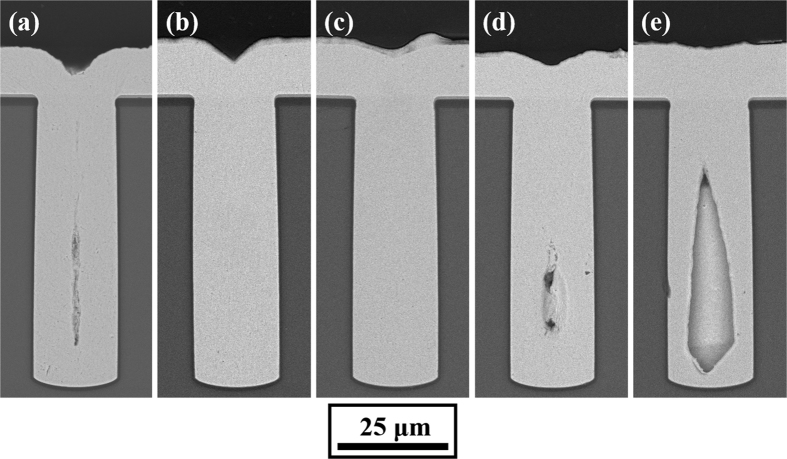
Cross-sectional SEM images of 20 × 65 μm blind-vias electrodeposited by direct current with different average current densities, (**a**) 4 mA/cm^2^, (**b**) 5 mA/cm^2^, (**c**) 7 mA/cm^2^, (**d**) 10 mA/cm^2^, and (**e**) 15 mA/cm^2^.

Figure 1 shows the cross-sectional SEM images for the TSV filling during electrodeposition at current densities of 4 mA/cm^2^, 5 mA/cm^2^, 7 mA/cm^2^, 10 mA/cm^2^, and 15 mA/cm^2^. According to Fig. 1, it can be seen that a change in the current density caused a change in the filling model. At a low current density (4 mA/cm^2^), a seam defect was observed at the centre of the via, as shown in Fig. 1(a). However, upon increasing the current density to 5 mA/cm^2^ and 7 mA/cm^2^, the TSVs were fully filled without any defects, as shown in Fig. 1(b) and (c). A further increase in the current density to 10 mA/cm^2^ and 15 mA/cm^2^ caused keyhole defects, which increased with current density, as shown in Fig. 1(d) and (e).

Therefore, there are mainly three kinds of filling models: low current density seam defect filling, medium current density defect-free filling, and high current density void defect filling. In industry, defect-free filling is the preferred result and seam defect filling is acceptable, but void defect filling should be avoided. Hence, a relatively low current density should be used to prevent voids, but this will entail a long deposition time and low throughput.

### Dynamic TSV filling process with low current density

**Figure 2:**
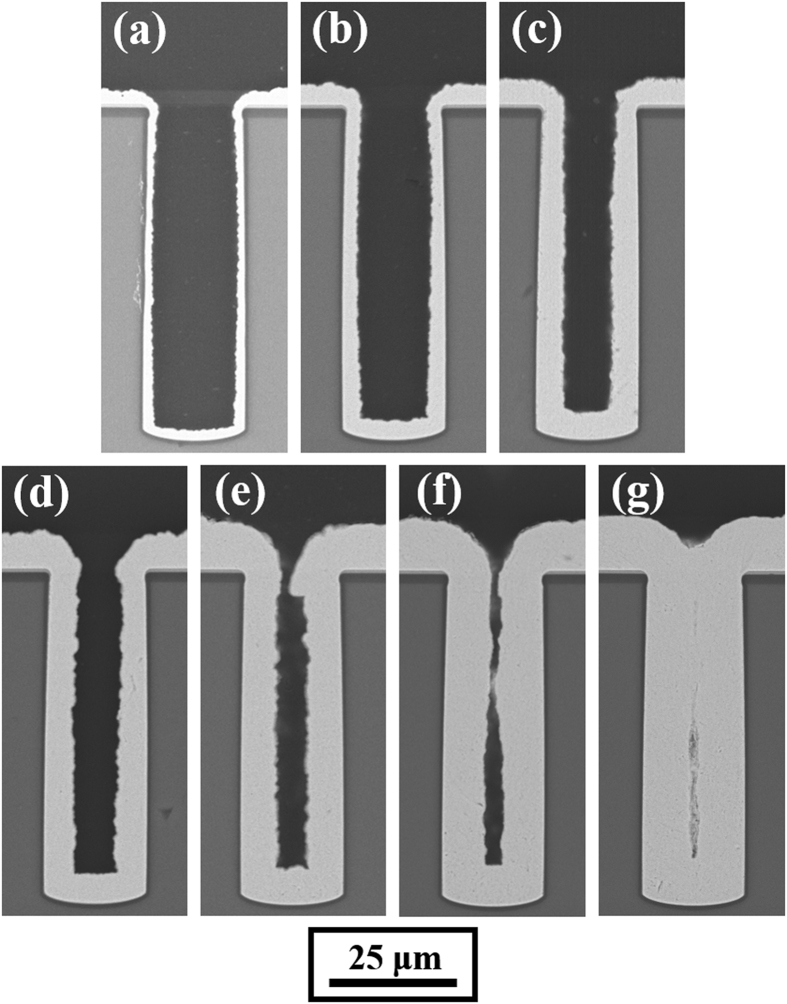
Cross-sectional SEM images of 20 × 65 μm blind-vias electrodeposited for the indicated periods of time with a constant average current density of 4 mA/cm^2^. The process of feature filling is captured at 30 min intervals: (**a**) 30 min, (**b**) 60 min, (**c**) 90 min, (**d**) 120 min, (**e**) 150 min, (**f**) 180 min, and (**g**) 210 min.

According to previous reports, a low current density results in a low deposition rate but seldom causes seam defects^12^. To understand how a seam defect is formed, experiments with deposition times of 30, 60, 90, 120, 150, 180, and 210 min were carried out at 4 mA/cm^2^. In this manner, the dynamic filling process could be observed clearly, as shown in Fig. 2.

The thicknesses of deposited copper on the bottom and on the sidewall are nearly the same in all cases, and bottom-up filling does is not observed in any of the cases. More precisely, during the first 90 min of electrodeposition, the plating thickness at the bottom was slightly greater than that at the top. However, during the last 120 min of electrodeposition, the plating thickness at the top gradually exceeded that at the bottom. This phenomenon finally caused a long seam in the centre of the via.

### Dynamic TSV filling process with medium current density

**Figure 3:**
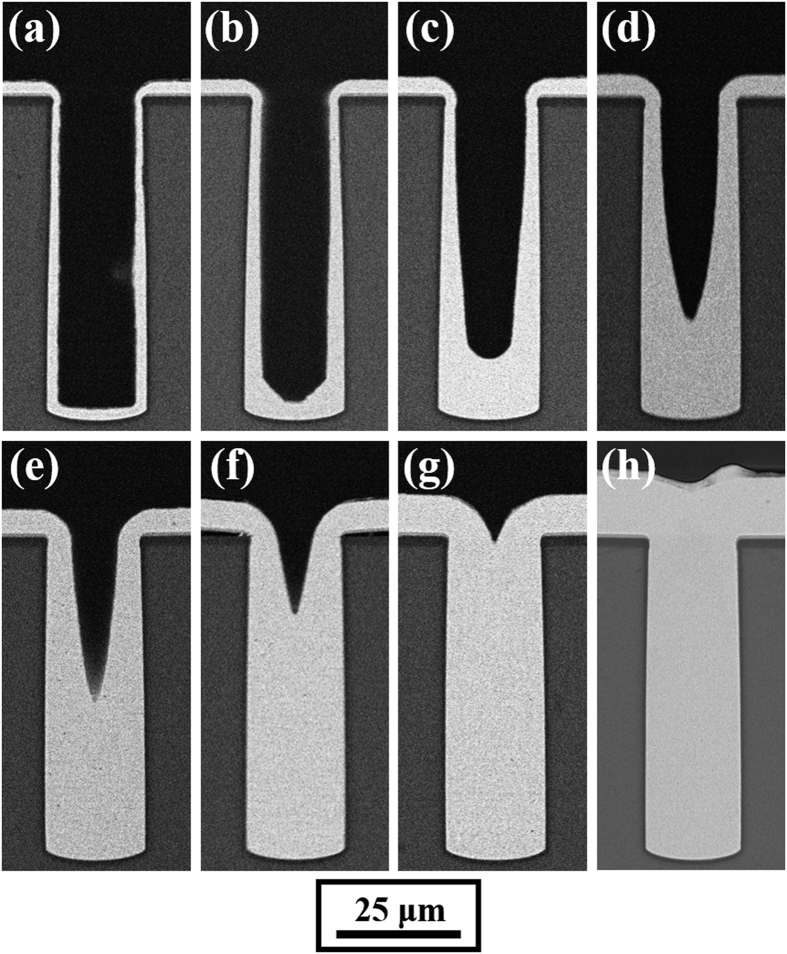
Cross-sectional SEM images of 20 × 65 μm blind-vias electrodeposited for the indicated periods of time with a constant average current density of 7 mA/cm^2^. The process of feature filling is captured at 10 min intervals: (**a**) 10 min, (**b**) 20 min, (**c**) 30 min, (**d**) 40 min, (**e**) 50 min, (**f**) 60 min, (**g**) 70 min, and (**h**) 80 min.

To further investigate the formation process of defect-free TSV filling, experiments with different deposition times from 10 to 80 min (at intervals of 10 min) were carried out. The current density was set at 7 mA/cm^2^. Figure 3 exhibits the cross-sectional SEM images of the experimental results.

As shown in Fig. 3(a), after 10 min electrodeposition, the thickness of the plating along the via surface was substantially equivalent, which resulted in a U-shaped profile. When the deposition time increased to 20 min, the bottom was filled slightly faster than the top; only the corner of the bottom was filled much faster than elsewhere as if the corner was chamfered, as shown in Fig. 3(b). After 30 min electrodeposition, the bottom was completely filled much faster than the top and the via was large at the top and small at the bottom, as shown in Fig. 3(c). After 40 min electrodeposition, faster deposition of the bottom resulted in a V-shaped via profile, as shown in Fig. 3(d). In the subsequent electroplating process, the morphology of the via remained V-shaped and the copper electrodeposition rate at the bottom of the via was higher than at the sidewalls, as shown in Fig. 3(e) to (h).

## Discussion

### Effect of current density on the TSV filling models

**Figure 4:**
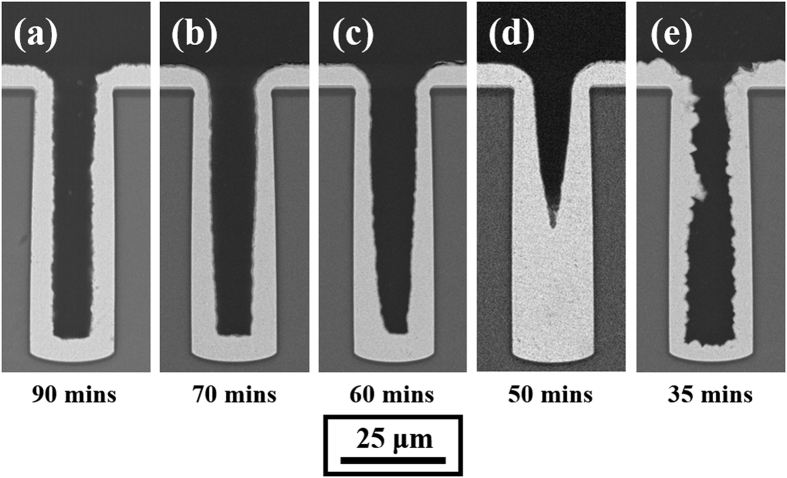
Cross-sectional SEM images of 20 × 65 μm blind-vias electrodeposited for the similar quantity of electric charge (Q = I × t) with different average current density: (**a**) 4 mA/cm^2^, (**b**) 5 mA/cm^2^, (**c**) 6 mA/cm^2^, (**d**) 7 mA/cm^2^, and (**e**) 10 mA/cm^2^.

**Figure 5:**
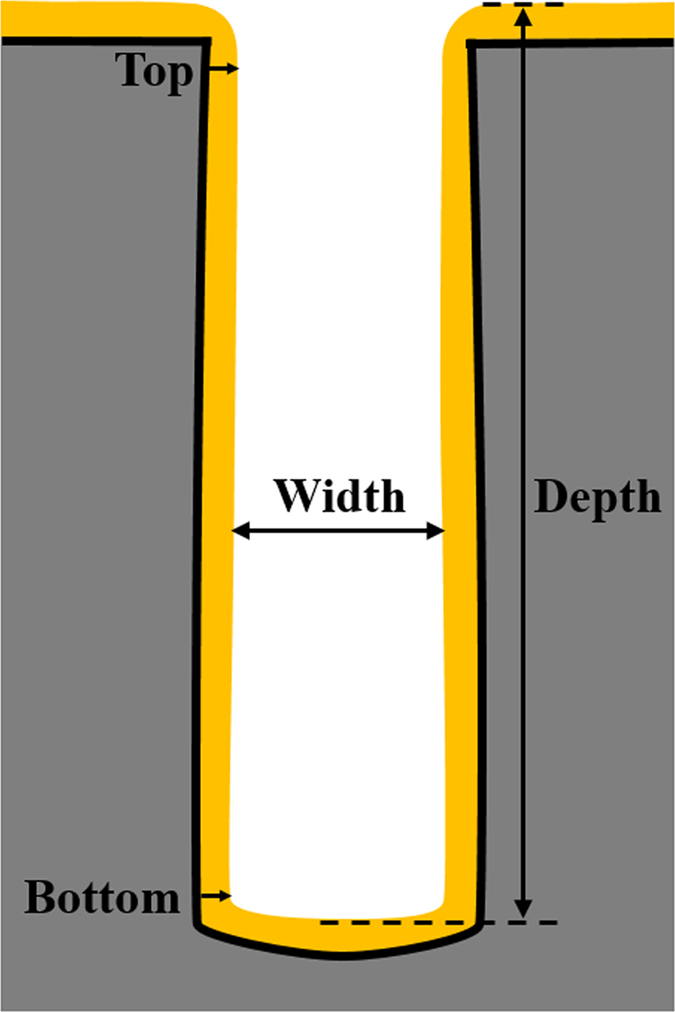
Schematic diagram for calculating the filling coefficient and the dynamic aspect ratio.

**Figure 6:**
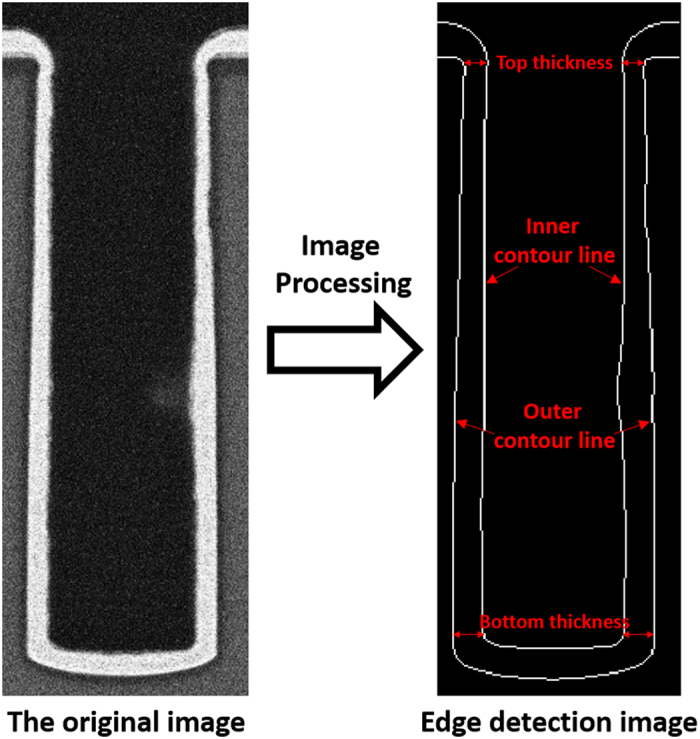
Method to measure the thickness of deposited copper.

**Figure 7:**
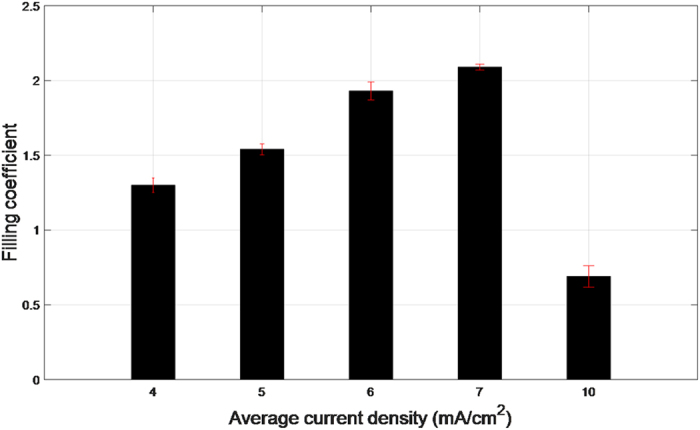
The filling coefficients of the staged TSV filling experiments carried out with different current densities.

Figure 4 shows the cross-sectional SEM images of the staged filling results for similar levels of electric charge (Q = I × t), when electrodeposition was performed at current densities of 4 mA/cm^2^, 5 mA/cm^2^, 6 mA/cm^2^, 7 mA/cm^2^, and 10 mA/cm^2^. It can be found that the thicknesses of deposited copper on the bottom and on the sidewall are different under different current density conditions. The ratio between the bottom and top thicknesses, as shown in Fig. 5, is defined as the filling coefficient to evaluate the filling model of TSV^13^. The thickness of copper deposited was calculated by image processing. As shown in Fig. 6, the contour of the plating was extracted from the original SEM image by the edge detection algorithm. The thickness of the plating was defined as the difference in pixel coordinates between the inner and outer contour lines. The top and bottom thicknesses were defined as the average of the right and left plating thicknesses at the top and bottom of the via, respectively. Further, 4 to 8 samples were measured for each data point to reduce the error. Figure 7 exhibits the filling coefficients of the staged TSV filling results shown in Fig. 4. The filling coefficient increased from 1.3 to 2.09 with an increase in current density from 4 mA/cm^2^ to 7 mA/cm^2^ and drastically reduced to 0.69 when the current density increased to 10 mA/cm^2^.

Therefore, the deposition rate at the bottom is slightly larger than that at the top under low current density conditions, and the difference in the deposition rate between the bottom and the top increases rapidly under medium current density conditions. However, the deposition rate at the bottom is lower than that at the top under high current density conditions.

These phenomena are caused by the dynamic change of the additives and copper ions. The overall deposition rate is controlled by the current density. Under low current density conditions, the overall deposition rate is low, and the copper ions and additives have sufficient time to diffuse to the bottom. Hence, the competitive adsorption between the accelerators and inhibitors is substantially uniform along the plating, and the copper ions are sufficient for the electrochemical reaction. These lead to an almost uniform deposition rate along the plating and result in conformal filling. However, with the increase in current density, the increased deposition rate accelerates the consumption of additives and copper ions. Due to their large molecular weight, the inhibitors diffuse to the bottom to a smaller extent than do the accelerators and copper ions. Hence, the inhibitors are gradually replaced by accelerators at the bottom. This causes the bottom deposition rate to be gradually higher than the top and results in super-conformal filling. Moreover, the concentration difference between the accelerators and inhibitors at the bottom increases with increasing current density until the diffusion of copper ions becomes slower than the electrochemical reaction. Therefore, the filling coefficient increases with the increase in current density within a certain range, as shown in Fig. 7. Under high current density conditions, the reaction of copper ions at the bottom could not be replenished in time due to the particularly high deposition rate. As a result, the deposition rate at the top is greater than at the bottom and results in sub-conformal filling.

### Effect of dynamic aspect ratio and curvature of plating on the local electrodeposition rate

The variation in plating morphology is caused by differences in the local electrodeposition rate. According to Fig. 3, at medium current density (7 mA/cm^2^), the maximum local deposition rate at the same stage is always located in the maximum curvature position. This phenomenon confirms the curvature enhanced accelerator coverage (CEAC) mechanism, which is that the local growth velocity is proportional to the curvature of the plating surface^25^,^26^,^27^. However, the CEAC theory is not suitable for the low current density condition.

**Figure 8:**
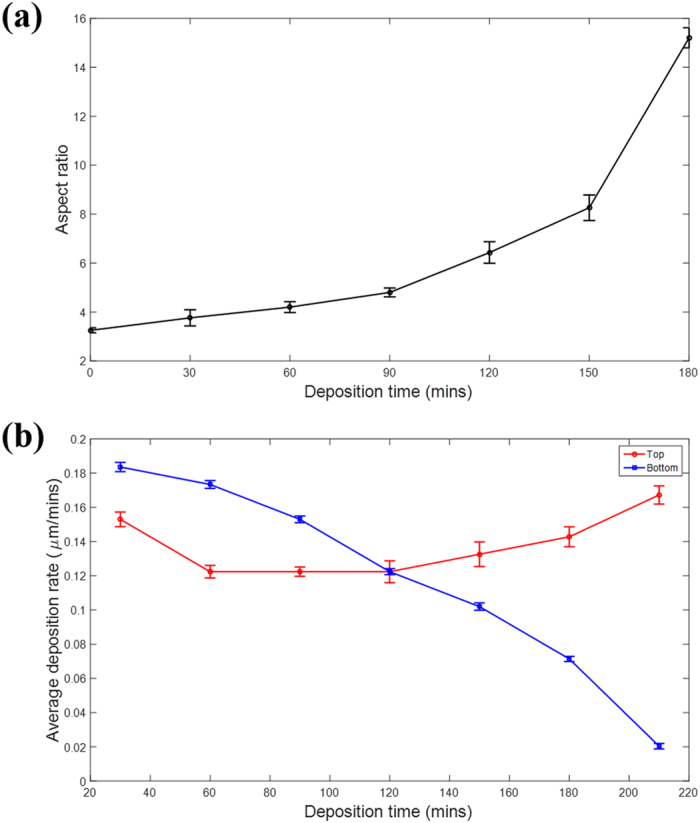
Effect of the dynamic aspect ratio on the local deposition rate in the low current density condition (4 mA/cm^2^). (**a**) A plot of the dynamic aspect ratio against electrodeposition time. (**b**) Plots of the deposition rate of the characteristic locations, *i.e*. the top and bottom on the sidewall of a via, against the electrodeposition time.

Figure 8 illustrates the effect of the dynamic aspect ratio on the local deposition rate in the low current density condition (4 mA/cm^2^). The dynamic aspect ratio is defined as the ratio between the depth and width, as shown in Fig. 5. The local electrodeposition rate is calculated by dividing the thickness difference of the plating electrodeposited at the adjacent moment by the time interval. As shown in Fig. 8(a), the aspect ratio of the via is 3.25 at the initial time. With the growth of the plating, the aspect ratio increased with the electrodeposition time. As shown in Fig. 8(b), the average deposition rate at the bottom decreased from 0.18 μm/min to 0.02 μm/min with the increase in aspect ratio. However, the average deposition rate at the top increased from 0.15 μm/min to 0.164 μm/min with increasing aspect ratio, except during the first 30 min.

The abnormal decline of the top deposition rate during the first 30 min was caused by the long rest time after pre-processing. Adequate diffusion of the copper ions and the additive molecules before the electrochemical reaction lead to relatively fast deposition in the initial stage. As the reaction progresses, copper ions in the electrolyte accept electrons from the cathode and are converted to copper continuously. As the aspect ratio increases, the diffusion rate of copper ions to the bottom decreases. The mass transport limitations on the cupric ion lower the rate of deposition to the bottom. Simultaneously, the accumulation of copper ions at the top improves the deposition rate. Gradually, the electrodeposition rate at the top exceeds that at the bottom and eventually results in the seam defect.

## Conclusions

Staged TSV filling experiments using the electrochemical deposition (ECD) method were conducted in this work at different current densities. Three representative morphologies were presented to describe the different filling results at different current densities. Additionally, the dynamic filling process was introduced to investigate the formation mechanisms of the typical filling results. The results indicate that low current density (4 mA/cm^2^) induces conformal filling with seam defects, medium current density (7 mA/cm^2^) induces super-conformal filling without defects, while high current density (10 mA/cm^2^) induces sub-conformal filling with void defects. Moreover, the influence of the applied current density on the TSV filling model was explained based on the synergistic mechanism of consumption and diffusion of additives and copper ions. Finally, the morphological evolution of plating reveals that the local deposition rate was affected by the geometrical characteristics of the plating, such as the aspect ratio and curvature, which is a new discovery in TSV filling research.

## Methods

### Plating solution

The electrolyte was made of 80 g/L CuSO_4_, 20 g/L MSA, 50 mg/L Cl^−^, 5 mL/L suppressor (ASTRI TSV-01 A), 5 mL/L accelerator (ASTRI TSV-01 B), and 7 mL/L levelling agent (ASTRI TSV-01 C), which were purchased from China Shenzhen Zhuoyuanbili Technology Co., Ltd.

### TSV chips

The TSV chips were purchased from Wuxi Huajin Semiconductor Co., Ltd. The size of the TSV chip was 10 mm × 10 mm. The TSV array consisted of 5 rows of vias with the pitch of 60 μm distributed along the chip edges. The size of the via in the TSV array was 65 μm × 20 μm.

### Instruments

**Figure 9:**
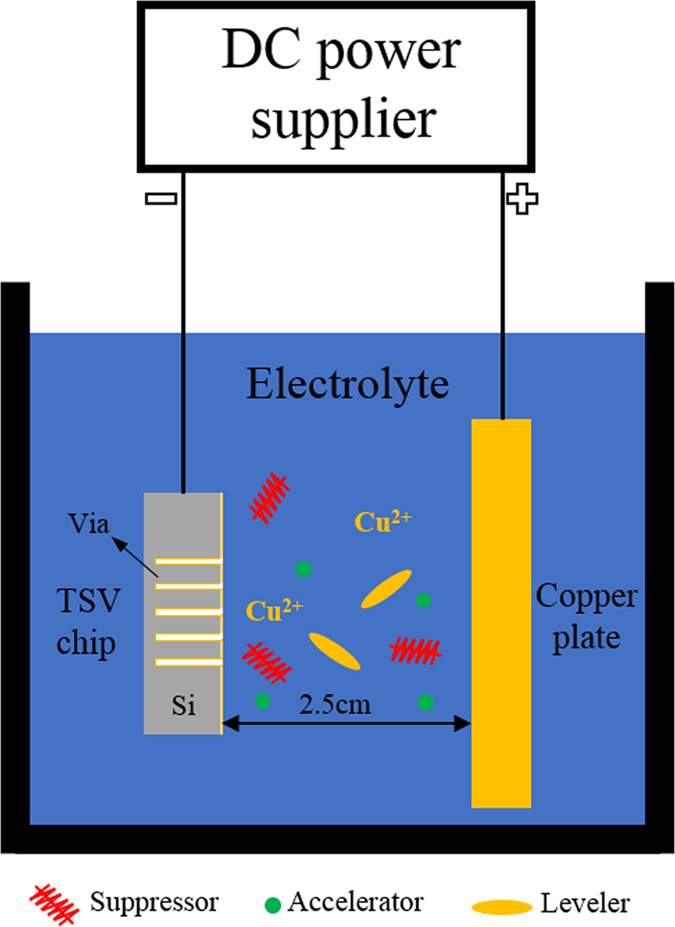
Schematic diagram of the experimental setup for TSV filling.

Electrodeposition was performed in a two-electrode configuration cell, as shown in Fig. 9. The anode was a copper plate (3 cm × 5 cm) and the cathode was a TSV chip. The distance between the anode and the cathode was 2.5 cm. The electrolyte cell with a volume of 300 mL was filled with 250 mL of fresh electrolyte.

### Pre-treatment

Before the electrodeposition process, the TSV chip was pre-treated to exclude air in the via and to wet the seed layer. First, the TSV chip was put into a suction bottle and immersed in deionized water. Then, the suction bottle was evacuated to a negative atmosphere using a water circulation pump. Under negative pressure, the air in the via was pushed into the sample piece surface. In addition, intermittent ultrasonic vibration was applied to remove the surface bubbles until no bubbles appeared, which indicates the completion of pre-treatment. Therewith, the TSV chip was moved to the plating bath rapidly and kept stationary for a sufficient time to ensure adequate diffusion of the plating solution within the via.

### Electrodeposition procedure

Following pre-treatment, the electrochemical deposition (ECD) process was carried out at different current densities (4 mA/cm^2^, 5 mA/cm^2^, 7 mA/cm^2^, 10 mA/cm^2^, and 15 mA/cm^2^) for the indicated periods of time. The electrodeposition intervals at the low current density condition (4 mA/cm^2^) and medium current density condition (7 mA/cm^2^) were 30 min and 10 min, respectively.

## Additional Information

**How to cite this article**: Wang, F. *et al*. Dynamic through-silicon-via filling process using copper electrochemical deposition at different current densities. *Sci. Rep.* **7**, 46639; doi: 10.1038/srep46639 (2017).

**Publisher's note:** Springer Nature remains neutral with regard to jurisdictional claims in published maps and institutional affiliations.
